# Structural Organization of Pregenomic RNA and the Carboxy-Terminal Domain of the Capsid Protein of Hepatitis B Virus

**DOI:** 10.1371/journal.ppat.1002919

**Published:** 2012-09-20

**Authors:** Joseph C.-Y. Wang, Mary S. Dhason, Adam Zlotnick

**Affiliations:** Department of Molecular and Cellular Biochemistry, Indiana University, Bloomington, Indiana, United States of America; Institut Pasteur, France

## Abstract

The Hepatitis B Virus (HBV) double-stranded DNA genome is reverse transcribed from its RNA pregenome (pgRNA) within the virus core (or capsid). Phosphorylation of the arginine-rich carboxy-terminal domain (CTD) of the HBV capsid protein (Cp183) is essential for pgRNA encapsidation and reverse transcription. However, the structure of the CTD remains poorly defined. Here we report sub-nanometer resolution cryo-EM structures of *in vitro* assembled empty and pgRNA-filled Cp183 capsids in unphosphorylated and phosphorylation-mimic states. In empty capsids, we found unexpected evidence of surface accessible CTD density partially occluding pores in the capsid surface. We also observed that CTD organization changed substantively as a function of phosphorylation. In RNA-filled capsids, unphosphorylated CTDs favored thick ropes of RNA, while the phosphorylation-mimic favored a mesh of thin, high-density strands suggestive of single stranded RNA. These results demonstrate that the CTD can regulate nucleic acid structure, supporting the hypothesis that the HBV capsid has a functional role as a nucleic acid chaperone.

## Author Summary

Many single stranded RNA virus encapsidate their genome through positively-charged domains of their capsid proteins. Hepatitis B virus (HBV) is a double stranded DNA virus which packages a single-stranded RNA pregenome (pgRNA) that is reverse transcribed within the capsid. RNA packaging requires a phosphorylated form of the HBV capsid protein's RNA-binding carboxy-terminal domain (CTD). Although the capsid has been well studied, the internal structures, the CTDs and the packaged RNA, are poorly characterized. By using in vitro reassembly, we have generated empty and pgRNA-filled capsids using phosphorylation-mimic and unphosphorylated forms of the capsid protein. Using cryo-EM image reconstruction, we have been able to show the structure of encapsidated pgRNA and, independently, the CTD in the absence of RNA to visualize early stages of the HBV assembly. We showed that the structural organization of the CTD changes as a function of the phosphorylation. Changes in CTD structure affect the structure of the encapsidated pgRNA, changing it from thin segments of density in the phosphorylated state, suggestive of single-stranded RNA, to thick rope-like structures consistent with duplex nucleic acid in the unphosphorylated state.

## Introduction

Chronic infection with hepatitis B virus (HBV) can lead to liver failure and cirrhosis; it is also the leading cause of hepatocellular carcinoma. Approximately 350 million individuals suffer from chronic HBV worldwide, and HBV contributes to 600,000 deaths per year [1]. It is a major public health issue and also a great social concern due to the discrimination against those infected with HBV in the endemic regions [2], [3].

HBV is an enveloped double-stranded (DS) DNA virus with an RNA intermediate form. In an infected cell, virion formation is initiated in the cytosol by the binding of a copy of the 3.2 kb “pregenomic” RNA (pgRNA) to the viral reverse transcriptase (RT) and packaging of the pgRNA• RT complex by multiple copies (180 or 240) of the viral capsid protein (Cp183) to form an immature core. Subsequently, the encapsidated single-stranded (SS) pgRNA is reverse transcribed to a full-length minus-stranded DNA that is covalently attached to the priming domain of RT; simultaneously, the RNA template is digested by the RNase H domain of the RT. A complementary, incomplete plus-strand DNA is then transcribed to generate rcDNA [4], [5]. These mature cores can then interact with viral surface proteins for envelopment. By studying the lifecycle of HBV we can identify new targets for development of antivirals as well as gain understanding of the function and behavior of these very specialized molecular machines.

The basic building block of the HBV capsid is the core protein homodimer. Most HBV cores are composed of 120 dimers arranged with T = 4 icosahedral symmetry; about 5% are 90-dimer T = 3 icosahedra [6]–[8]. The core protein has two domains: the N-terminal assembly domain (residues 1–149, which can be expressed as self-assembling Cp149) and the positively charged carboxy-terminal protamine-like domain (residues 150–183, CTD). The assembly domain forms the protein shell of the capsid [8]–[11]. The CTD is dispensable for capsid assembly but required for packaging RNA [10], [12]–[14], which it binds with high affinity [15]. Structures of empty T = 4, CTD-truncated Cp149 capsid have been solved to high resolution by cryo-EM and X-ray crystallography [9], [16], [17]. The Cp149 dimer has an overall shape of an inverted capital ‘T’ [8], [9], [18]. The stem of the ‘T’ is the four-helix bundle dimerization motif, which protrudes outward from the capsid surface. The crossbar of the ‘T’ clusters in a groups of five or six to form the contiguous capsid surface [8], [16]. Numerous pores perforating the capsid surface (located at the twofold (i.e. quasi-sixfold), threefold, and quasi-threefold axes) are proposed to allow nucleotides to diffuse in and out of the capsid during reverse transcription [9]. The CTD, localized to fivefold and quasi-sixfold vertices, extends into the capsid interior [18]–[20].

The capsid affects genome replication through its arginine-rich CTD. Phosphorylation of the CTD is important for RNA packaging and DNA synthesis [13], [21], [22]. The CTD has three SPRRR motifs (S155, S162, and S170) identified as the phosphorylation sites critical for pgRNA packaging [23], [24]. Mutation of these serines to alanine, to mimic the unphosphorylated capsid, suppresses pgRNA encapsidation [22], [25]–[27]. Replacing these serines with aspartate or glutamate to mimic phosphoserine supports pgRNA encapsidation but differentially affects transcription, suggesting that each repeat has an independent contribution to viral replication and may function together as a nucleic acid chaperone [21]. Although the identity of the enzyme involved in capsid phosphorylation is not clear, the serine/arginine-rich protein kinase (SRPK) family has demonstrated HBV kinase activity; SRPK1 physically binds to the CTD of both core homodimer and assembled capsid [15], [28], [29]. Such binding activity implies that the CTD resemble SR proteins, which are nuclear proteins involved in RNA splicing and transport from the nucleus. Indeed, core protein has substantial sequence identity with SR proteins [30]. Strikingly, though CTDs are on the inside of the capsid, the CTD also carries nuclear localization signals [31], suggesting that phosphorylation and accessibility of the CTDs can regulate intracellular trafficking of HBV cores [32], [33].

To test the hypothesis that phosphorylation of core protein CTDs has a structural role, we have determined cryo-EM structures of T = 4 HBV capsids assembled *in vitro* from unphosphorylated core protein (Cp183-SSS) or from a phosphorylation-mimic core protein (Cp183-EEE, carrying S155E, S162E, and S170E) with or without *in vitro* transcribed pgRNA. Our results clearly define the spatial location and the structural configuration of the CTDs and the encapsidated pgRNA. The CTD organization changes substantively as a function of phosphorylation state. This effect is transduced to the RNA structure. In unphosphorylated capsids, pgRNA formed an icosahedral cage that was virtually identical to the DS rcDNA in the native HBV virion, suggesting a largely DS state [34]. In the phosphorylation-mimic environment, the pgRNA formed a complicated mesh more consistent with RNA in a single-stranded state. This difference suggests that the HBV genome undergoes transient conformational changes during viral replication, which implies that the CTD is indeed a nucleic acid chaperone.

## Results

### Electron cryo-micrographs of empty particles and pgRNA-filled particles

**Figure 1:**
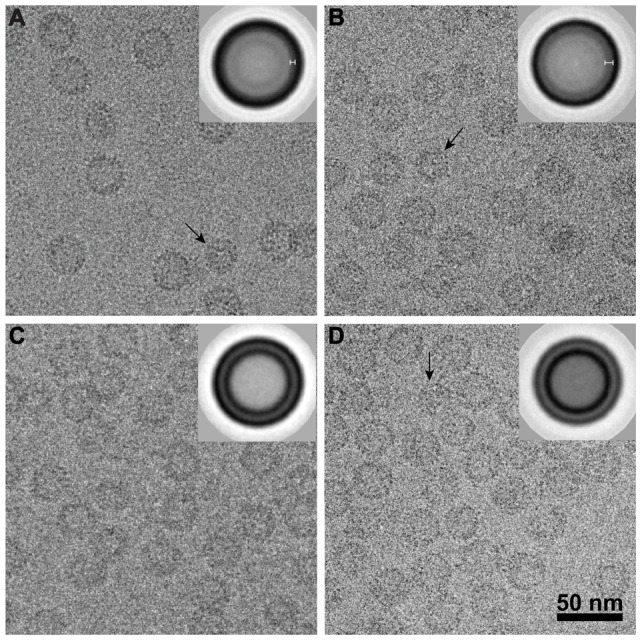
Cryo-micrographs of frozen-hydrated HBV capsids. (A) Cp183_e_-SSS (e for empty), (B) Cp183_e_-EEE, (C) Cp183_RNA_-SSS, and (D) Cp183_RNA_-EEE particles are shown, frozen hydrated in vitreous ice. These particles show the typical morphology of HBV capsids with characteristic spikes. These samples all have a minor population of smaller, T = 3 particles (black arrow). Inserts show translationally averaged images. Empty capsids (A, B) show a single ring corresponding to the protein shell; pgRNA-filled capsids (C, D) show two concentric rings, indicating the presence of an layer of nucleic acid. Note that the RNA ring in Cp183_RNA_-EEE is thicker than in Cp183_RNA_-SSS.

Purified HBV core protein dimers were reassembled into empty capsids (Cp183_e_-SSS and Cp183_e_-EEE) and pgRNA-filled capsids (Cp183_RNA_-SSS and Cp183_RNA_-EEE) [15]. RNA-filled capsids were dialyzed into buffered 150 mM NaCl while empty capsids were dialyzed into higher salt, 250 mM NaCl, to ensure stability [15]. Cryo-electron micrographs of Cp183_e_-SSS, Cp183_RNA_-SSS, Cp183_e_-EEE, and Cp183_RNA_-EEE (Figure 1) showed that all four constructs have similar morphology. pgRNA-filled particles noticeably had an inner layer of density characteristic of RNA-filled capsids [8], [18], [35]. To enhance the signal from the low contrast cryo-images, we translationally aligned particles to generate averaged images (Figure 1, insets). The empty Cp183_e_-SSS and Cp183_e_-EEE averages showed a single rim of density, 34 nm in diameter, indicating that they are both hollow spheres (Figure 1A and B). The averages of pgRNA-filled capsids (Figure 1C and D) showed an additional ring, presumably the encapsidated pgRNA; Cp183_RNA_-EEE appears to have much stronger RNA density than the Cp183_RNA_-SSS.

### Cryo-EM three-dimensional reconstructions

**Figure 2:**
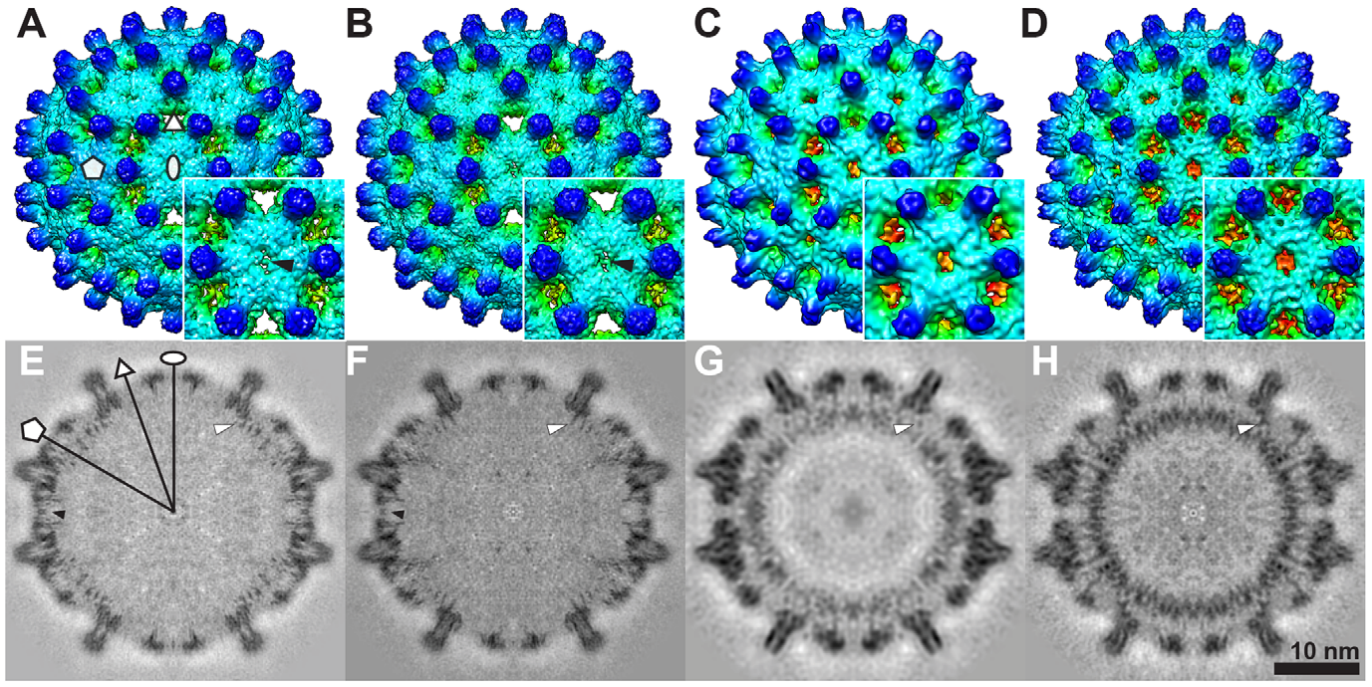
Cryo-EM 3D reconstructions of empty and pgRNA-filled Cp183 capsids. Surface shaded exterior maps of T = 4 (A) Cp183_e_-SSS, (B) Cp183_e_-EEE, (C) Cp183_RNA_-SSS, (D) Cp183_RNA_-EEE and their related central sections (E–H). Insets show enlarged views of the twofold (i.e. quasi-sixfold) vertex. All four maps have a similar external appearance with 120 spikes decorating a fenestrated capsid surface; the outer layer extends from a radius of 125 to 170 Å. In (A) Cp183_e_-SSS and (B) Cp183_e_-EEE, a thin layer of electron density partially occludes the central opening pore at the twofold axis (A, B, E, F, black arrows). This density is unique to the empty capsids. The pgRNA-filled capsids (C, D, G, H), on the other hand, lack the density across the twofold pore but display a substantial internal layer of density at the radii between 100–120 Å. In central sections (G, H), this density, corresponding to the co-assembled pgRNA, is clearly inhomogeneous indicating that the pgRNA has adopted a preferred conformation or constellation of conformations evident even though it has been icosahedrally averaged in these reconstructions. White arrows indicate the CTD tails tethered from the capsid inner surface. Oval, triangle, and pentagon indicate locations of twofold, threefold and fivefold axes, respectively.

To examine the details of pgRNA structure and the interaction between the capsid and pgRNA, we calculated image reconstructions of T = 4 particles to sub-nanometer resolution (Table 1). External views showed that all four types of particle (Cp183_e_-SSS, Cp183_e_-EEE, Cp183_RNA_-SSS, and Cp183_RNA_-EEE) were very similar (Figure 2A–D); nevertheless, from the central section it appeared that the spikes of the pgRNA-filled capsids adopted a slightly different quaternary structure (Figure 2E–F). As in previously published HBV structures [9], [16], [18], [34], [35], the capsids had pores at each twofold, threefold and quasi-threefold axis (Figure 2). Empty capsids, both Cp183_e_-SSS and Cp183_e_-EEE, appeared to have extra density partially occluding their twofold pores (i.e. quasi-sixfolds) (Figure 2A, B, E and F, black arrows). Presumably this density was from free CTDs. pgRNA-filled capsids (Figure 2C, D, G and H) and reconstructions of CTD-truncated particles [8]–[10], [16], [17] did not display this density. The central sections of the density maps showed short segments of density (Figure 2E–H, white arrows), presumably the CTDs, tethered from the capsid inner surface. In the empty Cp183_e_-SSS and Cp183_e_-EEE particles, this density was located under each dimer. Under the fivefold vertex, in Cp183_e_-EEE the CTD density condenses to form a funnel-like structure; in Cp183_e_-SSS, the equivalent density is weaker and forms distinct extensions.

**Table 1:** Image reconstruction data.

	Cp183_e_-SSS	Cp183_e_-EEE	Cp183_RNA_-SSS	Cp183_RNA_-EEE
**Number of particles (in reconstruction/total)**	27489/36676	14416/16967	7201/10502	7439/9196
**Number of CCD frames**	594	294	193	394
**Nominal magnification**	80,000×	80,000×	40,000×	80,000×
**Pixel size (Å)**	1.484	1.484	2.940	1.484
**Defocus ranges (µm)**	0.16–4.10	0.60–3.13	0.27–3.80	0.54–4.87
**Resolution (Å)**	5.5	5.8	8.0	7.0

Resolution was estimated based on a Fourier Shell Correlation of 0.5.

**Figure 3:**
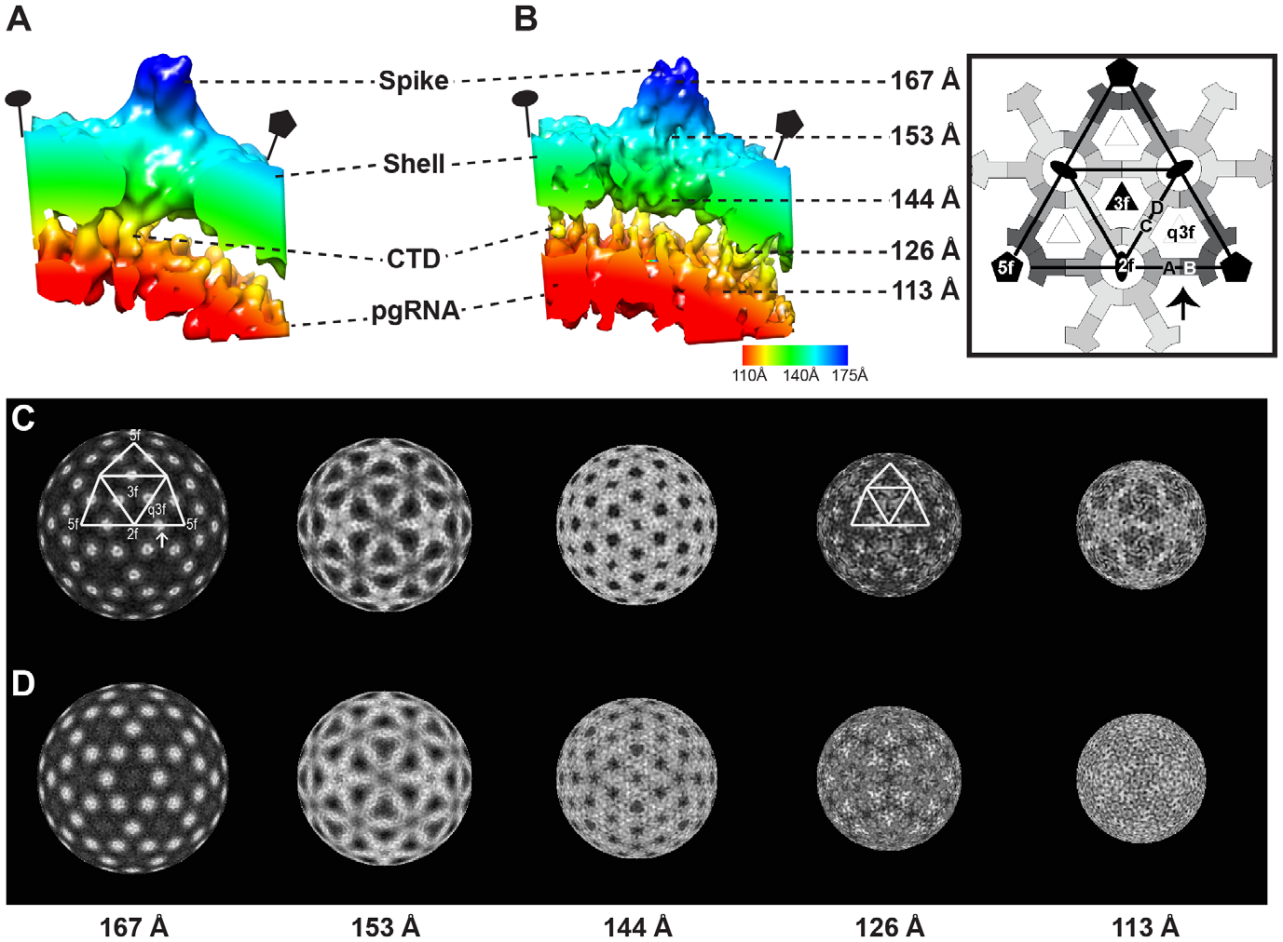
Interaction between the HBV capsid and pgRNA. A radially color-coded isosurface rendering of AB dimers and related pgRNA of (A) Cp183_RNA_-SSS and (B) Cp183_RNA_-EEE viewed from a 90° rotation of the region identified by an arrow in the rightmost inset. The Cp183_RNA_-EEE forms a massive pentagonal density under the fivefold vertex and correlating with a thicker layer of pgRNA than seen with the Cp183_RNA_-SSS reconstruction. Panels of radially cued densities of (C) Cp183_RNA_-SSS and (D) Cp183_RNA_-EEE, viewed along an icosahedral twofold axis at radii of 167, 153, 144, 126, 113 Å. In these images, the protein is presented in white with the high-to-low densities indicated by the gray scale. The density distribution patterns corresponding to capsid are very similar (three leftmost elements). CTDs are expected to be dominant features through radii of 117 to at 128 Å. At 126 Å the high-density features at CD dimer in Cp183_RNA_-SSS are tilted toward to the threefold axis, but the related density in Cp183_RNA_-EEE remains at the dimer position. Notably, the CTD density correlating with the A subunit is much weaker in the Cp183_RNA_-SSS, whereas Cp183_RNA_-EEE shows a strong propeller of density along the fivefold axes. At lower radius, pgRNA density shows distinct distributions. In the Cp183_RNA_-SSS, the density is strongest along the twofold edge connecting fivefold axis, which forms an icosahedral cage. In the Cp183_RNA_-EEE the density, while thinner than in Cp183_RNA_-SSS, forms a more evenly distributed sphere.

The differences between Cp183_e_-EEE and Cp183_e_-SSS CTD organization were also seen in pgRNA-filled particles. However, in RNA-filled capsids, density extending from the capsid inner surface submerged into the internal ring of RNA density (Figure 2G and H). In addition to the fivefold capsid-RNA connection, in Cp183_RNA_-SSS there were thin density elements connecting the extremities of the dimer to the RNA ring (Figure 3A). A different conformation for connecting density was observed at the Cp183_RNA_-EEE inner surface (Figure 3B).

The critical observation was that the RNA density in Cp183_RNA_-SSS and Cp183_RNA_-EEE adopts different ordered conformations. The strength and order of RNA density was observed in spite of the fact that the asymmetric RNA was subjected to icosahedral averaging (Figure 2G and H); if the RNA was not in part icosahedral, we would have expected a uniform shell of density. In Cp183_RNA_-SSS, RNA density was localized under twofold and fivefold vertices (Figure 2G); in Cp183_RNA_-EEE, the RNA density appeared as radially arrayed segments (Figure 2H). Radial density maps (Figure 3C and D) revealed similar core protein density distributions in Cp183_RNA_-SSS and Cp183_RNA_-EEE (at radii of 167, 153, 144 Å). However, the density shell at 113 Å showed that the RNA of Cp183_RNA_-SSS forms an icosahedral cage where fivefold pentamers are connected across twofolds; whereas in Cp183_RNA_-EEE, the RNA shell displayed a complicated mesh of density with the strongest density surrounding the fivefold vertices and comparatively weaker segments at the twofold vertices (Figure 3D).

A close comparison between Cp183_RNA_-SSS and Cp183_RNA_-EEE revealed the respective differences in the interactions between their CTDs and pgRNA (Figure 3A and B). In Cp183_RNA_-EEE, strong and continuous density originated from the A subunit and projected towards the fivefold axis and into the pgRNA density (Figure 3B). This stalactite-shaped density, also seen in the empty particle (compare Figure 2G to Figure 2H), is evident in the radial density map at radius of 126 Å (Figure 3D). Additional density, located under the CD dimer, connected down to the pgRNA layer at the quasi-threefold location. In contrast, the density connecting the inner capsid surface and the pgRNA in Cp183_RNA_-SSS was located directly under both AB and CD dimers. From the AB dimer, density projected straight into the pgRNA layer (Figure 3A and C); whereas in CD dimer, the density projected inward, obliquely toward the threefold axis and eventually immersed into pgRNA density (Figure 3C).

### Organization of the CTD in the HBV capsid

**Figure 4:**
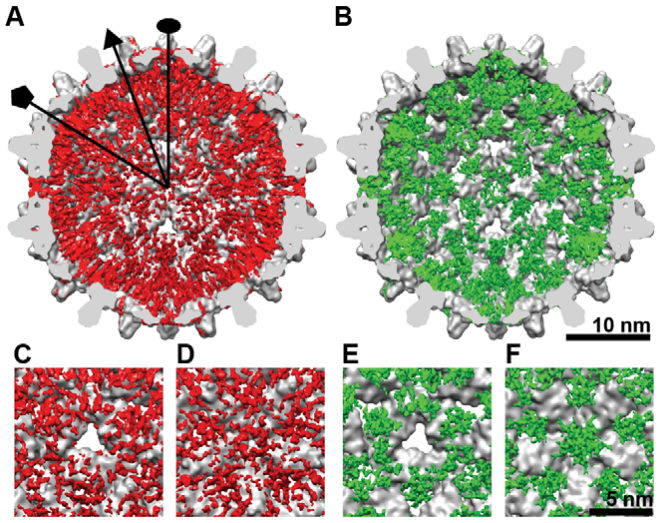
Spatial organization of the CTDs. Difference maps of CTD density were calculated by subtracting the low-pass filtered atomic model of Cp149 from the (A) Cp183_e_-SSS and (B) Cp183_e_-EEE. The resulting CTD density (red and green, respectively) was superimposed on the corresponding region of the interior of the Cp149 capsid. The bottom panels shows the enlarged views at the (C,E) threefold and (D,F) fivefold axes. The overall distributions of the CTDs in Cp183_e_-SSS and Cp183_e_-EEE are very similar except that the CTD density in Cp183_e_-SSS is more scattered whereas the CTD density in Cp183_e_-EEE forms a funnel-like shape under the fivefold vertex.

Difference maps (e.g. Cp183_e_-SSS less a molecular model of Cp149) revealed the overall CTD organization of empty HBV capsids in the unphosphorylated and phosphorylation-mimic states (Figure 4). Both maps were rendered at density levels needed to obtain the expected volume of the core protein (we note the signal was >1 σ). The locations of the CTDs within Cp183_e_-SSS (Figure 4A) and Cp183_e_-EEE (Figure 4B) shared a similar distribution, unsurprising as they erupt from the same regions of the contiguous capsid. However the organization and strength of the CTD density varied substantially. The CTD of the Cp183_e_-SSS formed five pillars of density surrounding the fivefold axis (Figure 4D), a density cluster under the quasi-threefold vertices (Figure 4C), and density that partially occluded the twofold opening.

**Figure 5:**
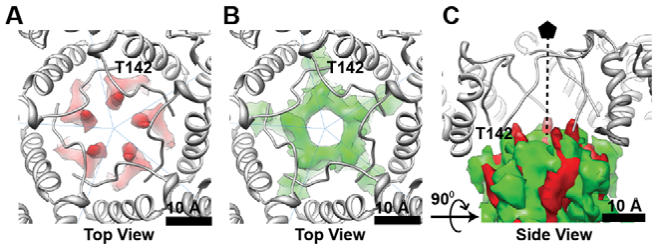
The phosphorylation-mimic EEE mutation alters CTD structure. Viewed from the capsid exterior, along a fivefold axis, the Cp149 atomic model (PDB entry 1QGT, gray) fits into cryo-EM density of (A, red) Cp183_e_-SSS and (B, green) Cp183_e_-EEE. The last visible residue of the crystal structure (T142) is close to the CTD difference density. When the CTD density of both Cp183 forms are overlaid (C, shows the superimposition side views of A and B), the movement of the peptide and the increased degree of interaction in Cp183_e_-EEE is immediately obvious, implying that the EEE mutation modulates a subunit-subunit interaction.

In Cp183_e_-EEE the fivefold CTD density, extending from each A subunit, formed a large stalactite-like structure extending to lower radius (Figure 4F). This structural characteristic was also observed in the Cp183_RNA_-EEE, where the fivefold stalactite density impinged on the pgRNA layer (Figure 3B and D at 126 Å). The last visible residue in the atomic model (Protein Data Bank (PDB) entry 1QGT) was close to the CTD density in both difference maps (Figure 5). However, the mutation of just three residues resulted in the shift of the CTDs from a relatively disordered structure in Cp183-SSS to a quaternary structure in Cp183-EEE that is suggestive of a convergence of five α-helices (See supporting information, Figure S1), one from each fivefold-related subunit. By comparison, the equivalent density in Cp183_e_-SSS is neither as strong nor cohesive; in fact, the overall CTD density in Cp183_e_-SSS was weaker and more scattered than in Cp183_e_-EEE.

CTD density was not quasi-equivalent. Unlike the CTD network observed at the quasi-threefold position, no CTD density was found beneath the threefold vertices (or twofold/quasi-sixfold vertices) in either Cp183_e_-SSS and Cp183_e_-EEE (Figures 3C and D, 4C and E).

### Structural comparison of pgRNA in Cp183_RNA_-SSS and Cp183_RNA_-EEE

**Figure 6:**
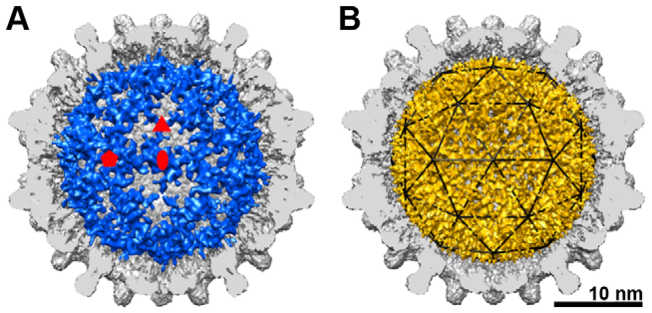
Structural organization of pgRNA. The difference maps of the pgRNA from (A) Cp183_RNA_-SSS (blue) and (B) Cp183_RNA_-EEE (gold) superimposed on cutaway of their respective empty capsids. By subtracting the empty Cp183 capsids from their respective pgRNA-filled capsids, the CTD-RNA interaction is evident as a gap, particularly evident on the fivefold of the EEE mutant (B). The pgRNA density in the unphosphorylated Cp183_RNA_-SSS forms an icosahedral cage, similar to the organization of rcDNA observed in the native virion. The pgRNA in the phosphorylation-mimic Cp183-EEE capsid shows a continuous mesh-like network density that appears to be composed of short segments of density.

Difference maps of the empty capsids subtracted from the pgRNA-filled capsids show that pgRNA adopted dramatically different structures in unphosphorylated and phosphorylated capsids (Figure 6). Density assigned to pgRNA in the Cp183_RNA_-SSS resembled an icosahedral cage (Figure 6A), closely matching the DS rcDNA structure found in the native (presumably unphosphorylated) virion [34]. The RNA density under the fivefold vertices was connected along the icosahedral twofolds. A branch of the RNA density extended from the twofold edge and terminated near the center of the threefold axis. The structural similarity between the pgRNA observed here and the dsDNA in the native virion, along with the thickness of the RNA density suggested that the icosahedrally ordered pgRNA in Cp183_RNA_-SSS may be largely DS RNA (Figures S2 and S3).

In striking comparison, the pgRNA in Cp183_RNA_-EEE formed a complicated mesh-like density network (Figure 6B, see Figure S2 for additional radial density maps). The pgRNA in Cp183_RNA_-SSS appeared to be condensed at the twofold position with a diameter of ∼20 Å consistent with double stranded nucleic acid. pgRNA in Cp183_RNA_-EEE resembled a net stretched over a sphere, where each segment in the mesh was approximately 7–8 Å thick, about the diameter of single-stranded RNA. Other analyses of the data supported this view. In the circularly averaged image (Fig. 1D) we observed that the pgRNA was stronger than capsid density. In the central cross-section of the three-dimensional map we observed that the pgRNA density was composed of short segments of strong density (Figure 2H). Furthermore, the Fourier shell correlation (FSC) at the radii corresponding to the pgRNA shell (90–118 Å) indicated a resolution of 6 Å at the 0.5 cutoff (Figure S4), which is slightly better than the overall resolution estimated for the whole 3D model (7 Å at FSC 0.5 cutoff). These metrics indicated that the pgRNA within Cp183_RNA_-EEE had substantial structural order. We suggest that the conformational rearrangement of the pgRNA between expanded and condensed forms depends on Cp183 phosphorylation state.

## Discussion

In this study, we determined the 3-D structures of the CTD and pgRNA of *in vitro* assembled HBV particles using cryo-EM and 3-D image reconstruction. Based on cell culture studies [22], [25], [26], we used Cp183-EEE to mimic the effects of phosphorylation. Through difference map imaging, subtracting x-ray coordinates of an HBV capsid of C-terminally truncated capsid proteins [9] from selected Cp183 capsids, we found that the inclusion of the EEE mutation in the CTDs had profound effects on the CTD structure. The altered CTD organization resulted in an equally dramatic reorganization of packaged pgRNA.

### Conformational changes of the CTD and RNA associated with phosphorylation

The phosphorylation state of HBV is believed to change during the course of assembly and reverse transcription. The initial assembly reaction involves a phosphorylated form of the core protein [36], [37]. In a related hepadnavirus, duck hepatitis B virus, the immature phosphorylated capsid becomes hypophosphorylated as it matures [36], [37]. Dephosphorylation likely occurred at the point when the plus-stranded DNA was synthesized [38], [39]. The core protein in DNA-filled cores was unphosphorylated [36], [37].

Our 3-D reconstructions of empty Cp183_e_-SSS and Cp183_e_-EEE show that the structures of the HBV assembly domains (residues 1–149) were similar to existing crystal structures and followed T = 4 quasi-equivalence (Figure S5). However, the SSS and EEE CTDs had very different conformations. For both Cp183_e_-SSS and Cp183_e_-EEE, CTD density emerged from the capsid near the last visible residue in the atomic coordinates (Figures 4 and 5). The CTD density in Cp183_e_-EEE appeared to be more compact (Figure 4A and B). The five independent pillars surrounding the fivefold vertex of Cp183_e_-SSS may be the result of the electrostatic repulsion from the positively charged CTDs (Figure 5A and C). It was anticipated that the positively charged CTD in Cp183_e_-SSS would be mobile, resulting in weak, incomplete density. We were surprised to find that the three negative charges of the Cp183-EEE mutant resulted in much greater order (Figure 5B and C). The relatively strong density of the fivefold stalactites suggested formation of a tightly folded complex. Typically, compact folds are stabilized by a hydrophobic core, but in this case we suggest that the organization of these complexes is supported by salt bridges. This result leads us to speculate that regulatable salt bridges have a similar structural role in SR proteins, which also require phosphorylation for activity. No similar density formation was observed beneath the quasi-sixfold vertex. It is possible that the larger opening at the quasi-sixfold pore provides extra space to increase CTD mobility.

**Figure 7:**
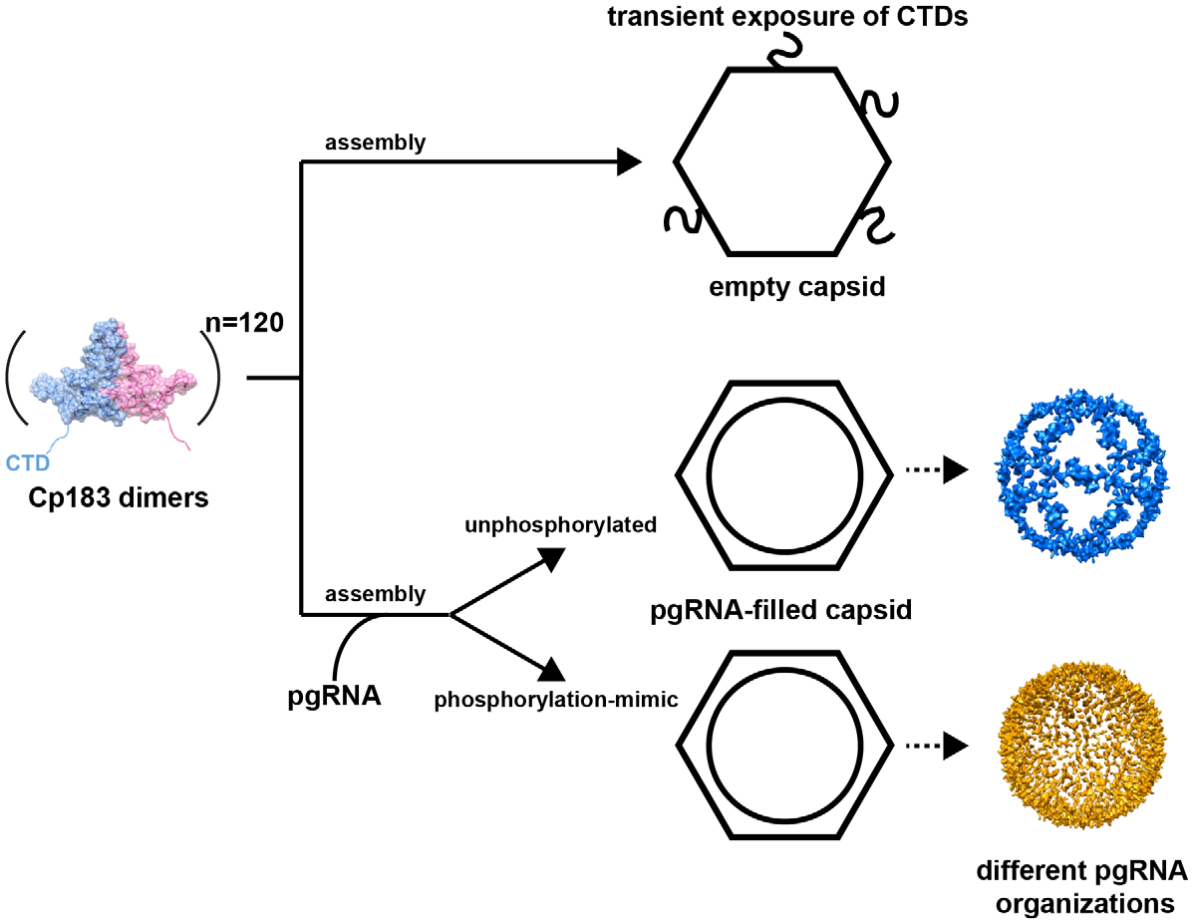
An assembly schema for HBV Cp183 capsids. Assembly of Cp183 dimers in the absence of RNA results in empty capsids with CTDs transiently exposed through the pores on the icosahedral twofold axes. Co-assembly of Cp183 dimers with pgRNA results in RNA-filled capsids where the RNA structure is responsive to the phosphorylation states of the CTD.

The distinct structures of the encapsidated pgRNA in Cp183_RNA_-SSS and Cp183_RNA_-EEE suggest a novel response by RNA to the phosphorylation state of hepadnavirus core protein (Figure 7). The pgRNA density in Cp183_RNA_-SSS shares a common organization with the nucleic acid density observed in rcDNA-filled particles (from transgenic mice and human sources) and virus-like particles containing RNA from an *E. Coli* expression system [8], [34]. These disparate particles both show an icosahedral cage of nucleic acid stretching from fivefold to fivefold. The structural similarity suggests that the nucleic acid was organized by the CTDs. Conversely, the RNA density in the Cp183_RNA_-EEE capsid was visibly stronger in both the 2-D averaged image (Figure 1D, inset) and the central section of the 3-D reconstruction (Figure 2H).

So, what is the basis of the difference in the strength of the RNA density? We cannot rule out the possibility that some empty capsids may be accidently selected in Cp183-SSS or Cp183-EEE reconstructions though these were not prevalent in sucrose gradient experiments (Figure S6). The pgRNA density in Cp183_RNA_-SSS is similar to previously published HBV structures with unphosphorylated core protein where the RNA density is always weaker than capsid density [8], [18], [34], even where the amount of encapsidated RNA is about the size of a genome [18]. In surface representations (using a density cutoff based on the capsid volume, Figure 6), the calculated volumes of the pgRNA in Cp183_RNA_-EEE and Cp183_RNA_-SSS were about the same, 1.1×10^6^ Å^3^, a volume consistent with the 3.2 kb pgRNA assuming an average RNA density of 1.7 g•cm^−3^. This suggests both particles encapsidated the same amount of pgRNA. Indeed, sucrose gradient velocity sedimentation suggested that the majority of the Cp183_RNA_-SSS capsid contain one pgRNA (Figure S6). Based on charge, Cp183-EEE has less capacity for RNA binding than Cp183-SSS; electrophoretic mobility shift titrations of RNA by Cp183-EEE saturated only when there was sufficient protein for one RNA per capsid [15]. To test for differences in RNA order in our reconstructions, we examined the volume of the RNA density at different contour levels. We found that the relative pgRNA volume in Cp183_RNA_-SSS decreased faster than in Cp183_RNA_-EEE; the ratio of RNA volume to capsid volume is shown in Table S1. Thus, we suggest that the difference in the strength of the RNA density is due to the relative disorder of RNA in Cp183_RNA_-SSS.

The difference in RNA order appears to correlate with differences in structure. In the Cp183_RNA_-SSS structure, the surface shaded RNA density was thick enough to accommodate DS RNA. In Cp183_RNA_-EEE the narrow strands of density could only fit single-stranded RNA (Figures 6B, S2 and S3). We suggest that a single-stranded pgRNA structure is more favorable for reverse transcription. The ability of the CTD to affect RNA structure was consistent with the hypothesis that the core protein itself (via the CTDs) can act as an RNA chaperone [21], [40]. The correlation between dephosphorylation of the CTDs [36] and reverse transcription remains to be elucidated. However, the responsiveness of RNA to CTD phosphorylation (i.e. SSS versus EEE) state and the observed progressive changes in phosphorylation [21], [22], [25], [27], [36], [39] suggest that the pgRNA structures of Cp183_RNA_-SSS and Cp183_RNA_-EEE shown here are only two of many possible structures along a conformational continuum.

Thus, our data indicate that the CTDs change conformation in response to phosphorylation and transduce a conformational change in the packaged nucleic acid. We propose that these changes are linked to reverse transcription. A similar modulation on the nucleic acid structure by reversible phosphorylation has also been reported for histone H1 protein, which contains SPXX repeats (where X can be K or R) that are similar to the SPRRR motifs found in the HBV CTD [41], [42]. The phosphorylation states of the histone H1 protein seem to affect its secondary structure and are involved in both condensation and decondensation of the chromatin at different stages during the process of DNA replication [43]–[46]. While the Cp183_RNA_-EEE is a novel structure, the pgRNA in the Cp183-SSS capsid resembles previous unphosphorylated structures containing RNA and DNA. The conformational similarity between the pgRNA in Cp183_RNA_-SSS and previous rcDNA structures, as well as the dimensions of the pgRNA density, suggests that at least part of the single-stranded pgRNA is condensed into a duplex architecture. It is notable that the ability of a virus capsid to control the structure of packaged RNA has also been observed in Pariacoto virus and flock house virus, even with non-native RNA [47], [48]. Thus, our findings suggest that the conformation of the icosahedrally arrayed CTDs and the packaged nucleic acid represents a mobile compromise of electrostatics and the equilibrium and non-equilibrium thermodynamics that is vital to virus function.

### Functional implication of the CTD surface exposure in negatively regulating the signal for the viral envelopment

Although the majority of the density corresponding to the CTD was found internally at the quasi-sixfold, threefold and fivefold vertices (Figure 4A and B), it has been long postulated that the CTD is partially exposed to the capsid surface for the purpose of signaling during viral replication [9], [28], [49]–[51]. Our results (here and previously [28]) suggest that the CTD may be accessible through the large quasi-sixfold pore (Figure 7). We observed CTD-attributed density passing through the quasi-sixfold pore in empty capsids (Figure 2A and B inserts, black arrows); similar density has not been observed in CTD-truncated or genome-filled HBV capsids. Indeed, probing empty Cp183_e_-SSS capsids with the CTD-specific SRPK results in the capsid decorated by SRPK at every quasi-sixfold vertex [28]. The failure of capsids filled with *E. Coli* RNA to bind to SRPK columns [28] indicates that CTDs are not readily accessible when associated with RNA.

Ning et al. suggested that single-stranded nucleic acid (either pgRNA or single-stranded DNA) in the immature capsids negatively regulates HBV core trafficking by preventing CTD exposure [52]. They observed that secreted enveloped particles contained either empty capsids (over 90%) or DS rcDNA-filled cores. Thus, the blocking hypothesis predicts that mature DS rcDNA-containing capsid shares structural characteristics with the empty capsid [52]. This hypothesis is supported by EM reconstructions. First, in empty capsids, the CTDs were partially exposed through the quasi-sixfold pore (Figure 2E and F). Second, in the presence of the pgRNA, CTDs strongly interacted with the genome; the single-stranded genome obstructed exposure of CTD-associated signals [28]. Third, the partially DS rcDNA in the mature core is expected to be much less flexible and may not be able to interact with all of the CTDs, which suggests that a fraction of the CTDs may regain their mobility in mature DS rcDNA cores [53]. Thus, nucleic acid-regulated exposure of CTDs through quasi-sixfold pores is a likely mechanism for signaling by the HBV core.

In summary, we report sub-nanometer resolution structures of the full-length empty and pgRNA-filled HBV capsids assembled from unphosphorylated and phosphorylation-mimic core proteins. The structures show that the configurations of the RNA-binding CTDs and pgRNA respond to changes in CTD phosphorylation. Our data indicate that phosphorylation affects the structure of CTDs and the CTDs affect RNA organization. Such functional correlation of the CTD implies that the HBV core has nucleic acid chaperone activity. We further provide direct evidence of partially exposed CTDs on the capsid exterior, suggesting how they may play a role in intracellular trafficking and secretion of HBV cores. Even though we cannot draw a complete structural description of HBV maturation yet, the substantial changes of the CTD and pgRNA we observed in this study indicated that HBV is a highly dynamic molecular machine. Nevertheless, in the authentic capsid the viral RT, and possibly host factors, take critical parts in pgRNA packaging; their impact on the structure of encapsidated pgRNA is currently under investigation.

## Materials and Methods

### Purification of HBV capsids

The pgRNA production, the plasmids coded for HBV Cp183-SSS and Cp183-EEE, and the capsid purification were described previously [15]. Capsids stored at −80°C were disassembled by dialysis at 4°C in the disassembly buffers (1.5 M guanidine HCl, 0.5 M LiCl, 50 mM HEPES at pH 7.5, 10 mM DTT for Cp183-SSS, and 1.5 M guanidine HCl, 1.5 M LiCl, 50 mM Tris at pH 9.5, 10 mM DTT for Cp183-EEE). The encapsidated heterogeneous RNA packaged from *E. Coli* cells was precipitated by a spin of 20,000× g for 15 min at 4°C. Protein dimers were recovered from the supernatant and purified by size exclusion chromatography using an analytical grade Superose 6 column (GE Lifesciences) equilibrated in disassembly buffer. Fractions containing core protein were identified by SDS-PAGE. Cp183-SSS and Cp183-EEE dimers were either used for the reassembly experiments immediately or stored at 4°C for a short period. Formation of the empty capsids (Cp183_e_-SSS and Cp183_e_-EEE) was approached by dialyzing the purified dimers in the reassembly buffer (250 mM NaCl, 50 mM HEPES at pH 7.5, 2 mM DTT for Cp183-SSS and 250 mM NaCl, 50 mM Tris pH 7.4, 2 mM DTT for Cp183-EEE). The pgRNA-filled capsids (Cp183_RNA_-SSS and Cp183_RNA_-EEE) were prepared by reassembling the purified dimers with *in vitro* transcribed HBV pgRNA at a molar ratio of protein dimer to RNA polymer = 120∶1 in the reassembly buffer (150 mM NaCl, 50 mM HEPES at pH 7.5, 2 mM DTT for Cp183-SSS and 150 mM NaCl, 50 mM Tris at pH 7.4, 2 mM DTT for Cp183-EEE) overnight. Samples for cryo-EM were further concentrated by Amicon Ultra centrifugal filter units (Millipore, MA). The quality and the concentration of the sample were routinely checked by negative stained EM using 2% uranyl acetate.

### Cryo-electron microscopy

The sample preparation and cryo-EM operation were followed well established procedures described previously [28]. Briefly, a drop of 3.5 µl sample solution was applied on a glow-discharged Quantifoil holey-carbon grid (R2/2), blotted with filter paper from both sides for 4 s to produce a thin layer of specimen solution across the holes. The grids were quickly plunged into liquid ethane bath cooled by liquid nitrogen in a cryo-container. All processes described above were performed by a FEI Vitrobot. The vitrified specimen on the grid was then transferred to a Gatan 626DH cryo-holder and kept at the low temperature environment (<−176°C) for the subsequent processing. The cryo-holder was then rapidly inserted into a JEOL-3200FS EM (JEOL Ltd., Japan) operated at 300 kV with an in-column energy filter using a 20-eV slit except for Cp183_RNA_-SSS. Digitized images were recorded under the low-dose condition (<20 e^−^/Å^2^) on an UltraScan 4000 4k×4k CCD camera (Gatan Inc., Oxford, UK) at a nominal magnification of 80,000× (equal to 0.1484 nm at the specimen space) for Cp183_e_-SSS, Cp183_e_-EEE, and Cp183_RNA_-EEE and 40,000× (equal to 0.294 nm at the specimen space) for Cp183_RNA_-SSS. Images were taken at multiple defocuses to compensate the effect from the contrast transfer function of the EM.

### Image processing

Selected images which fulfilled the criteria of the suitable particle concentration, optimal ice thickness and minimal specimen drift, were used for analysis. Particle images were semi-automatically boxed using program e2boxer.py from EMAN2 software [54]. The defocus level of each micrograph was estimated using RobEM (http://cryoem.ucsd.edu/programs-old.shtm) and only the phase reversal was corrected in the subsequent data processing. The initial starting model for each specimen was reconstructed by an *ab initio* random model method [55]. Origin and orientation were determined and refined using AUTO3DEM [56]. The refinement processed iteratively with a successively improved 3-D model from each refinement until a stable 3-D reconstruction had been achieved. The resolution of each 3-D reconstruction was estimated by Fourier shell correlation using a threshold value of 0.5. The final 3-D maps of Cp183_e_-SSS, Cp183_e_-EEE, Cp183_RNA_-SSS and Cp183_RNA_-EEE were reached at the resolutions of 5.5 Å, 5.8 Å, 8.0 Å, and 7.0 Å, respectively (Figure S7). The 3-D reconstructions were visualized using RobEM and Chimera [57].

### Difference map analysis

A CTD-truncated HBV capsid, Cp149_model_, was calculated from the atomic coordinates (Protein Data Bank entry: 1QGT) using e2pdb2mrc.py and low-pass filtered to 10 Å. The difference map of CTD was calculated by subtracting Cp149_model_ from Cp183_e_-SSS or Cp183_e_-EEE; the difference map of pgRNA was calculated by subtracting Cp183_e_-SSS from Cp183_RNA_-SSS or Cp183_e_-EEE from Cp183_RNA_-EEE. Prior to the subtraction, all maps were normalized based on their average density and the standard deviation because the map calculated from the crystal structure generally has different density values than the cryo-EM reconstruction. The sizes of the maps were scaled, and the difference observed here was less than 1% in all cases. The region corresponding to the capsid shell (at the radii between 125–160 Å) was then used to calibrate the density. The resulting difference map was rendered at the contour level that is equivalent to that component rendered at the estimated full mass of the parental 3-D model.

### Accession codes

The cryo-EM density maps have been deposited to EMDataBank.org. The EMDataBank accession number for Cp183_e_-SSS, Cp183_e_-EEE, Cp183_RNA_-SSS, and Cp183_RNA_-EEE are EMD-2057, EMD-2058, EMD-2059, and EMD-2060, respectively.

## Supporting Information

Figure S1 **The 5-fold stalactite-like density in Cp183_e_-EEE reveals the potential five α-helices conformation.** Inside-out view (left) and side view (right) of the modeled α-helix (gray, in the ribbon representation) fitted into the stalactite-like CTD density of Cp183_e_-EEE (transparent green isosurface) at the fivefold vertex. Red star points the α-helix structure. Pentagon indicates the fivefold axis.
(TIF)

Figure S2 **Selected radial sections of pgRNA density in unphosphorylated and phosphorylation-mimic reconstructions.** Color-coded radial sections of the difference maps of pgRNA in the Cp183_RNA_-SSS capsid (left column) and the Cp183-EEE capsid (right column). Areas used for the close-up views are marked by the rectangle. In Cp183-SSS the blue color is the density value used for the isosurface rendering in Figure 6A and the green color is used to show the region where the density level greater than 4σ (4 standard deviations above the mean value). In Cp183_RNA_-EEE the red color is the density value used for the isosurface rendering in Figure 6B and the yellow color is to show the density level greater than 4σ.
(TIF)

Figure S3 **Model RNA nucleotide into cryo-EM density map of pgRNA.** Single-stranded and/or double-stranded RNA nucleotides docked into the cryo-EM difference maps of pgRNA calculated from (A) Cp183_RNA_-SSS and (B) Cp183_RNA_-EEE viewed along icosahedral twofold axis. The backbone of DS RNA is rendered in a ribbon representation. The total numbers of modeled nucleotides are 3000 nucleotides for Cp183_RNA_-SSS and 3060 nucleotides for Cp183_RNA_-EEE, respectively.
(TIF)

Figure S4 **Radial density profiles and resolution assessments of pgRNA-filled capsids.** Normalized radial density profiles (solid lines) of the reconstructed Cp183_RNA_-SSS (blue) and Cp183_RNA_-EEE (orange) capsids showed the characteristic density profile representing the capsid with protruding spikes and the pgRNA. Two radial resolution curves (dash lines, using FSC cutoff at 0.5) for the Cp183_RNA_-SSS (blue) and Cp183_RNA_-EEE (orange) reconstructions show the resolution variation as a function of radius. The radial FSC was assessed using 15-Å-thick shells from radii of 0 to 195 Å.
(TIF)

Figure S5 **Fitting of Cp149 X-ray model into cryo-EM reconstruction of Cp183_e_-SSS.** (A) The crystal structure of the assembly domain (PDB 1QGT, in the ribbon representation) was fitted into 5.5-Å cryo-EM density map (transparent gray isosurface) of Cp183_e_-SSS as one rigid body. Each subunit is shown in different color (A in red, B in yellow, C in blue, and D in green). Zoomed stereo pairs of the (B) AB dimer and (C) CD dimer viewed from the corresponding direction are indicated by the arrows in (A). Oval, triangle, and pentagon indicate locations of twofold, threefold and fivefold axes, respectively.
(TIF)

Figure S6 **Sucrose gradient analysis of Cp183_RNA_-SSS.** Reassembled Cp183_RNA_-SSS capsids were initially centrifuged at 15,000× g for 15 mins at 4°C to remove large aggregates. The resulting supernatant was layered onto linear 10–60% sucrose gradients in 500 mM NaCl, 50 mM HEPES at pH 7.5 and centrifuged in a Beckman SW40Ti rotor at 39,000 rpm (190,000× g) for 2 h at 4°C. Fractions were manually collected from the bottom and assayed by an HPLC system equipped with a diode array UV-vis detector (Shimadzu) using a Bio SEC-5 HPLC column with a 500 Å pore diameter (Agilent). Light scattering-corrected UV absorbances at 260 nm, 280 nm, and the corrected 260 nm/280 nm ratios were calculated [58] and plotted for each fraction. Selected fractions were analyzed by negative stained EM using 2% uranyl acetate. The results showed a major peak containing unaggregated particles (Fractions 21–28) and a minor peak (Fractions 19–20) containing aggregated particles. In further analysis of the major peak we found that the fractions near the top of the gradient (Fractions 25–28) contained a mixture of T = 3 and T = 4 particles. Based on absorbance, the average nucleotide per dimer in this region was about 32, suggesting a mixture of 33% of T = 4 particles and 67% of T = 3 particles; although some empty capsids could also have sedimented in this region. Fractions 21–24 contained mainly T = 4 particles and the calculated average of 27 nucleotides per dimer, or 3240 nucleotides per T = 4 capsid, suggested that there was one pgRNA per capsid.
(TIF)

Figure S7 **Resolution estimation of 3-D reconstructions.** A FSC plot for different reconstructions. The resolution cutoff value of 0.5 is identified with the dashed line.
(TIF)

Table S1 **Relative volume calculation in Cp183_RNA_-SSS and Cp183_RNA_-EEE.** The estimated volume was calculated using the “Measure Volume and Area” function in Chimera. The pgRNA volume was measured for the difference maps of pgRNA calculated from Cp183_RNA_-SSS and Cp183_RNA_-EEE, respectively. To accomplish this calculation, the contour level of the whole particle (capsid+pgRNA) was first rendered at a contour that accommodated 100% of the expected mass and then the volume of the pgRNA in the difference map was adjusted to match the pgRNA in the whole particle. The same procedure was applied to calculate the volume rendered at 50% of the expected mass. In the 50% mass calculation, if both capsid and pgRNA were perfectly ordered, one would expect 7.6% pgRNA by volume. The results showed that only 2.8% of the volume was contributed by the pgRNA in Cp183_RNA_-SSS whereas 6.1% of the volume was contributed by the pgRNA in Cp183_RNA_-EEE. The faster disappearance of the pgRNA in Cp183_RNA_-SSS compared to Cp183_RNA_-EEE suggested that the pgRNA in Cp183_RNA_-SSS is more disordered though a similar result could occur if the dataset was contaminated by a large fraction of empty particles.
(DOCX)
