# Supplementary figures and images for: Structural Organization of Pregenomic RNA and the Carboxy-Terminal Domain of the Capsid Protein of Hepatitis B Virus
Source: PLoS Pathog. 2012 Sep 20;8(9):e1002919. doi: 10.1371/journal.ppat.1002919 (PMC3447754; doi:10.1371/journal.ppat.1002919)

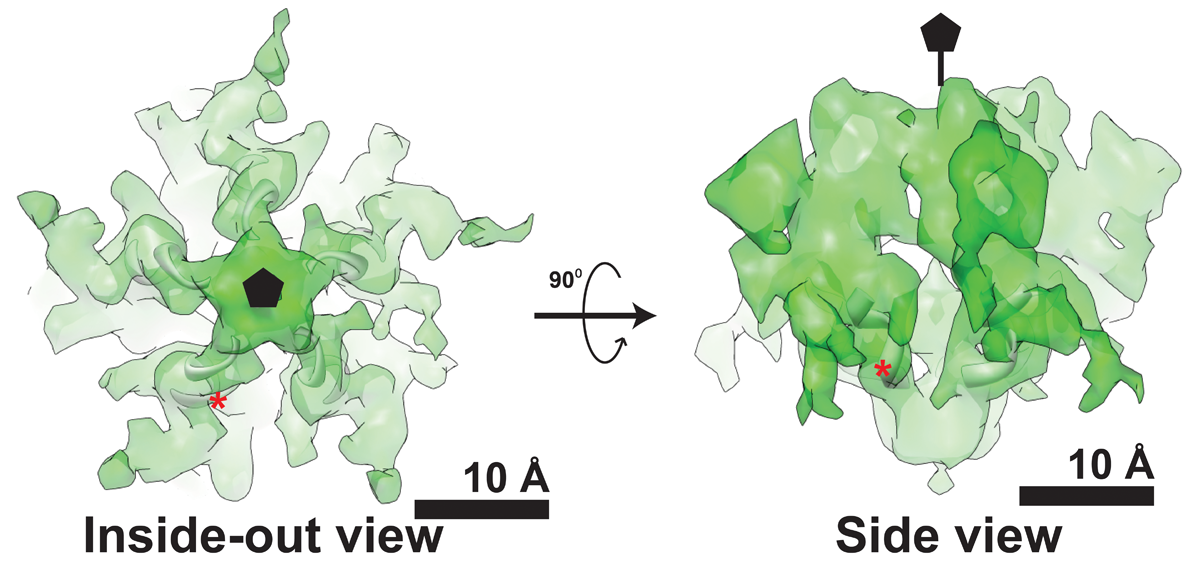

Supplement: Figure S1 — The 5-fold stalactite-like density in Cp183e-EEE reveals the potential five α-helices conformation. Inside-out view (left) and side view (right) of the modeled α-helix (gray, in the ribbon representation) fitted into the stalactite-like CTD density of Cp183e-EEE (transparent green isosurface) at the fivefold vertex. Red star points the α-helix structure. Pentagon indicates the fivefold axis. (TIF) [file ppat.1002919.s001.tif]

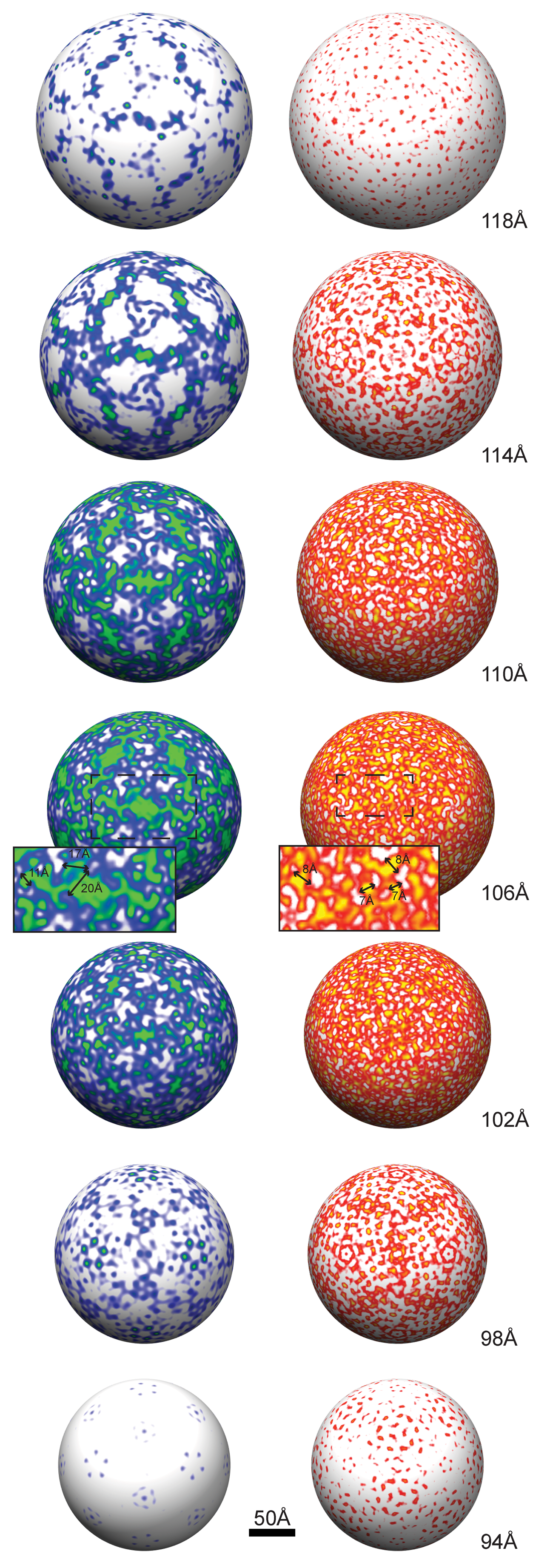

Supplement: Figure S2 — Selected radial sections of pgRNA density in unphosphorylated and phosphorylation-mimic reconstructions. Color-coded radial sections of the difference maps of pgRNA in the Cp183RNA-SSS capsid (left column) and the Cp183-EEE capsid (right column). Areas used for the close-up views are marked by the rectangle. In Cp183-SSS the blue color is the density value used for the isosurface rendering in Figure 6A and the green color is used to show the region where the density level greater than 4σ (4 standard deviations above the mean value). In Cp183RNA-EEE the red color is the density value used for the isosurface rendering in Figure 6B and the yellow color is to show the density level greater than 4σ. (TIF) [file ppat.1002919.s002.tif]

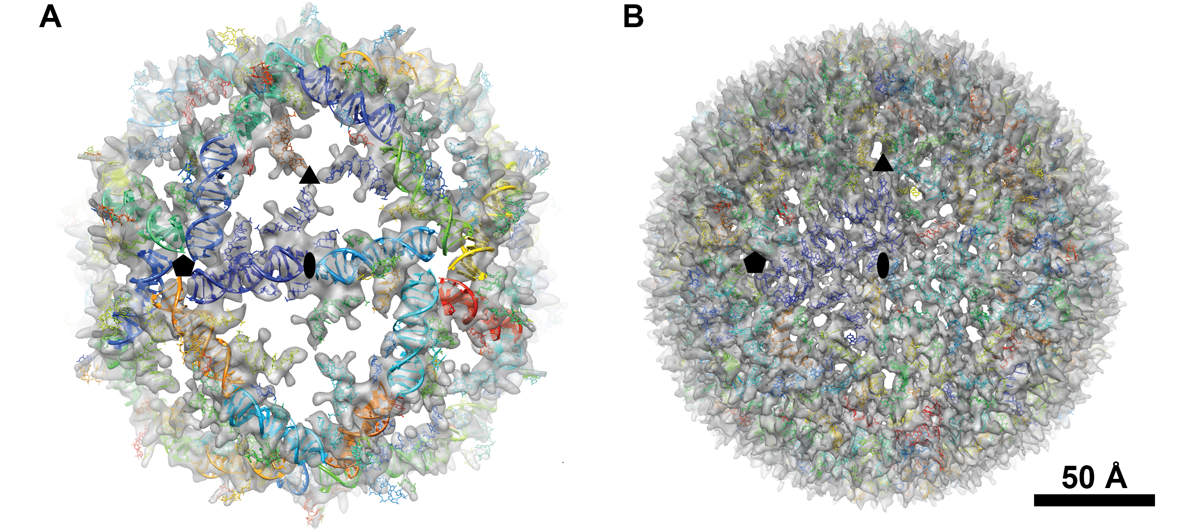

Supplement: Figure S3 — Model RNA nucleotide into cryo-EM density map of pgRNA. Single-stranded and/or double-stranded RNA nucleotides docked into the cryo-EM difference maps of pgRNA calculated from (A) Cp183RNA-SSS and (B) Cp183RNA-EEE viewed along icosahedral twofold axis. The backbone of DS RNA is rendered in a ribbon representation. The total numbers of modeled nucleotides are 3000 nucleotides for Cp183RNA-SSS and 3060 nucleotides for Cp183RNA-EEE, respectively. (TIF) [file ppat.1002919.s003.tif]

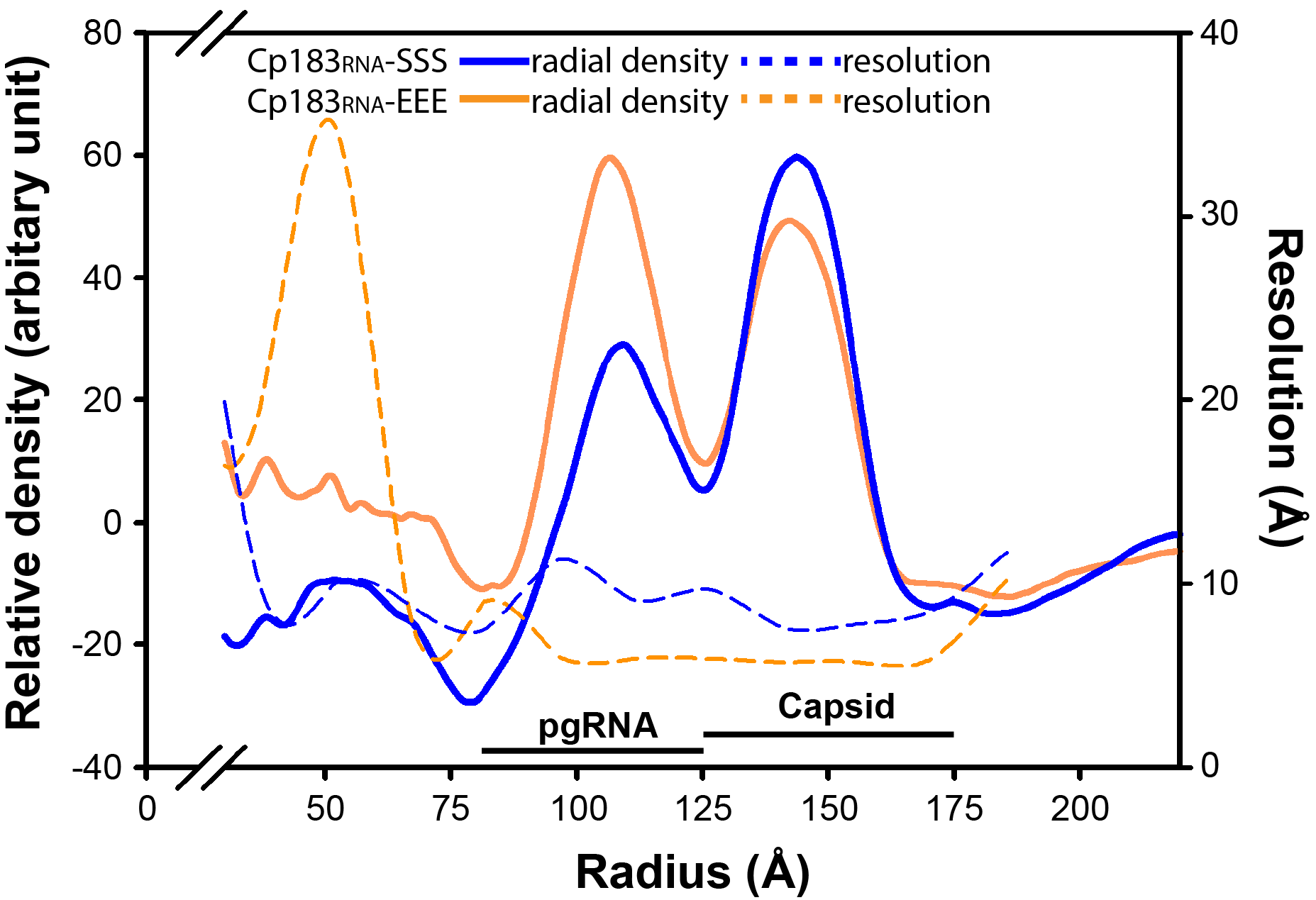

Supplement: Figure S4 — Radial density profiles and resolution assessments of pgRNA-filled capsids. Normalized radial density profiles (solid lines) of the reconstructed Cp183RNA-SSS (blue) and Cp183RNA-EEE (orange) capsids showed the characteristic density profile representing the capsid with protruding spikes and the pgRNA. Two radial resolution curves (dash lines, using FSC cutoff at 0.5) for the Cp183RNA-SSS (blue) and Cp183RNA-EEE (orange) reconstructions show the resolution variation as a function of radius. The radial FSC was assessed using 15-Å-thick shells from radii of 0 to 195 Å. (TIF) [file ppat.1002919.s004.tif]

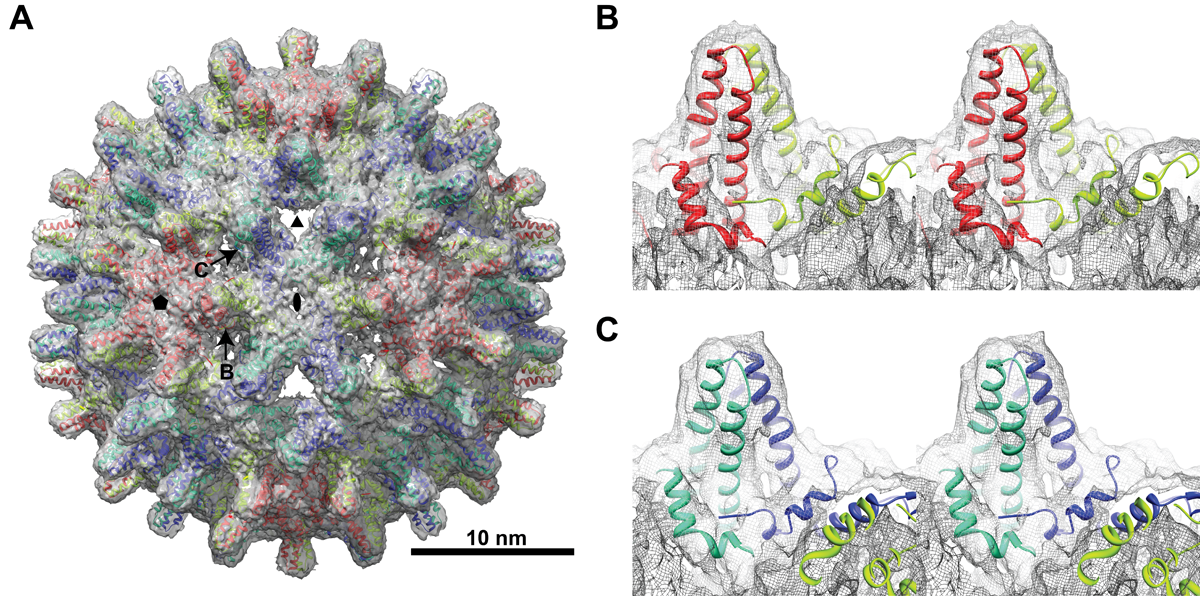

Supplement: Figure S5 — Fitting of Cp149 X-ray model into cryo-EM reconstruction of Cp183e-SSS. (A) The crystal structure of the assembly domain (PDB 1QGT, in the ribbon representation) was fitted into 5.5-Å cryo-EM density map (transparent gray isosurface) of Cp183e-SSS as one rigid body. Each subunit is shown in different color (A in red, B in yellow, C in blue, and D in green). Zoomed stereo pairs of the (B) AB dimer and (C) CD dimer viewed from the corresponding direction are indicated by the arrows in (A). Oval, triangle, and pentagon indicate locations of twofold, threefold and fivefold axes, respectively. (TIF) [file ppat.1002919.s005.tif]

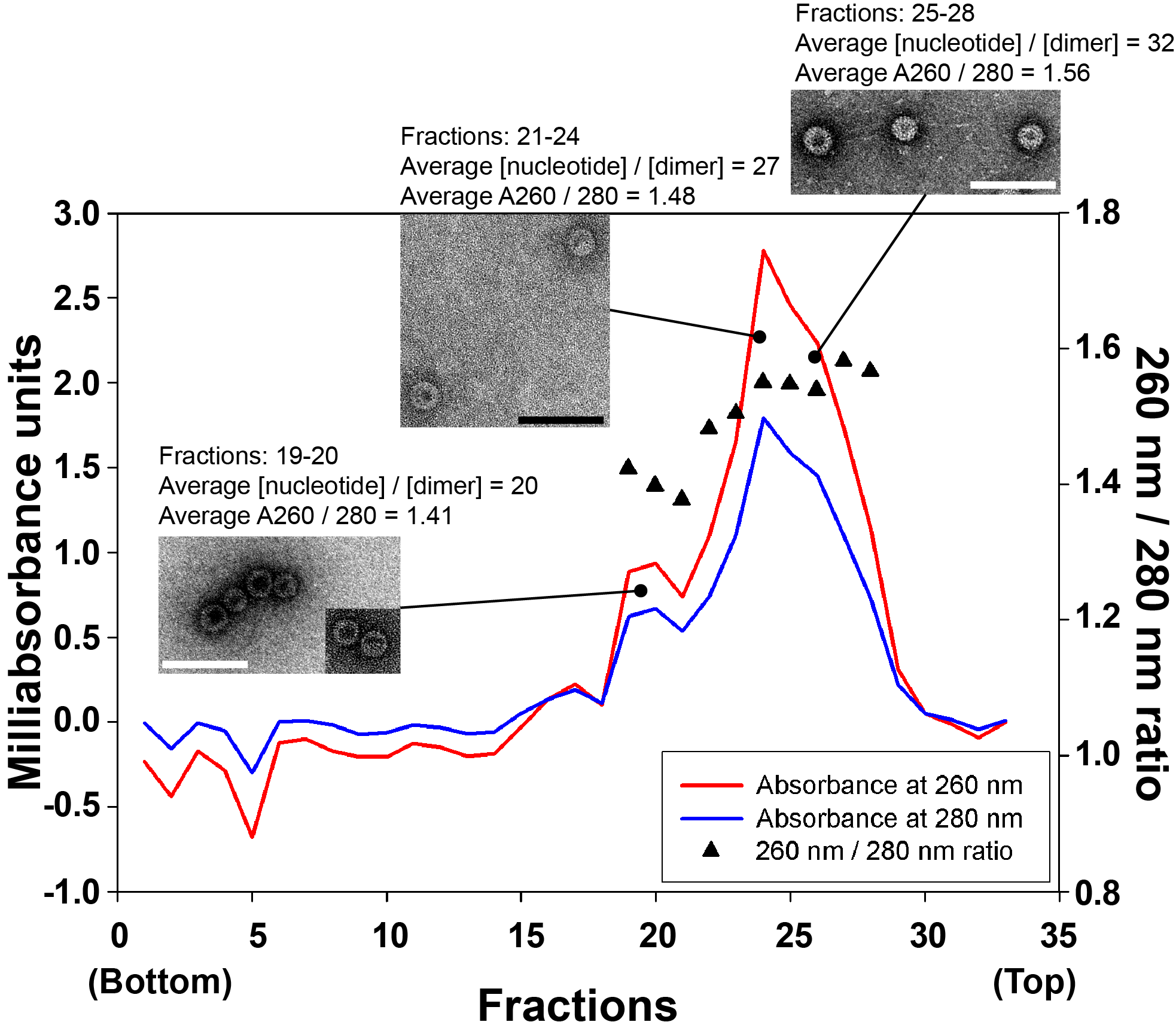

Supplement: Figure S6 — Sucrose gradient analysis of Cp183RNA-SSS. Reassembled Cp183RNA-SSS capsids were initially centrifuged at 15,000× g for 15 mins at 4°C to remove large aggregates. The resulting supernatant was layered onto linear 10–60% sucrose gradients in 500 mM NaCl, 50 mM HEPES at pH 7.5 and centrifuged in a Beckman SW40Ti rotor at 39,000 rpm (190,000× g) for 2 h at 4°C. Fractions were manually collected from the bottom and assayed by an HPLC system equipped with a diode array UV-vis detector (Shimadzu) using a Bio SEC-5 HPLC column with a 500 Å pore diameter (Agilent). Light scattering-corrected UV absorbances at 260 nm, 280 nm, and the corrected 260 nm/280 nm ratios were calculated [58] and plotted for each fraction. Selected fractions were analyzed by negative stained EM using 2% uranyl acetate. The results showed a major peak containing unaggregated particles (Fractions 21–28) and a minor peak (Fractions 19–20) containing aggregated particles. In further analysis of the major peak we found that the fractions near the top of the gradient (Fractions 25–28) contained a mixture of T = 3 and T = 4 particles. Based on absorbance, the average nucleotide per dimer in this region was about 32, suggesting a mixture of 33% of T = 4 particles and 67% of T = 3 particles; although some empty capsids could also have sedimented in this region. Fractions 21–24 contained mainly T = 4 particles and the calculated average of 27 nucleotides per dimer, or 3240 nucleotides per T = 4 capsid, suggested that there was one pgRNA per capsid. (TIF) [file ppat.1002919.s006.tif]

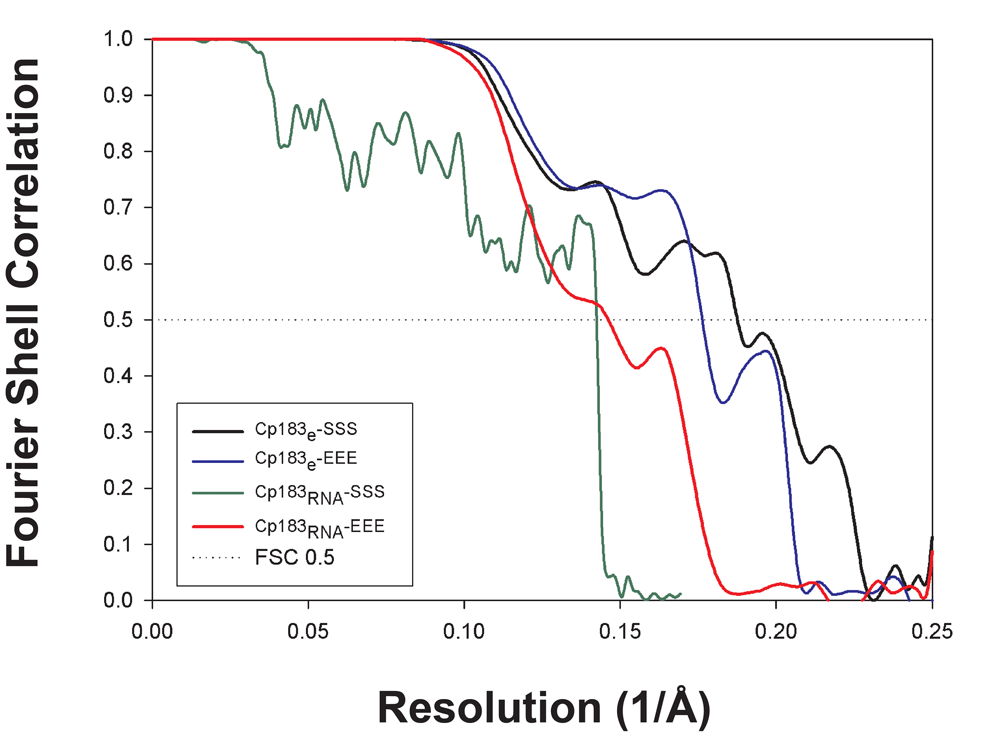

Supplement: Figure S7 — Resolution estimation of 3-D reconstructions. A FSC plot for different reconstructions. The resolution cutoff value of 0.5 is identified with the dashed line. (TIF) [file ppat.1002919.s007.tif]
