# Supplementary material for: Structural Organization of Pregenomic RNA and the Carboxy-Terminal Domain of the Capsid Protein of Hepatitis B Virus
Source: PLoS Pathog. 2012 Sep 20;8(9):e1002919. doi: 10.1371/journal.ppat.1002919 (PMC3447754; doi:10.1371/journal.ppat.1002919)
Supplement: Table S1 — Relative volume calculation in Cp183RNA-SSS and Cp183RNA-EEE. The estimated volume was calculated using the “Measure Volume and Area” function in Chimera. The pgRNA volume was measured for the difference maps of pgRNA calculated from Cp183RNA-SSS and Cp183RNA-EEE, respectively. To accomplish this calculation, the contour level of the whole particle (capsid+pgRNA) was first rendered at a contour that accommodated 100% of the expected mass and then the volume of the pgRNA in the difference map was adjusted to match the pgRNA in the whole particle. The same procedure was applied to calculate the volume rendered at 50% of the expected mass. In the 50% mass calculation, if both capsid and pgRNA were perfectly ordered, one would expect 7.6% pgRNA by volume. The results showed that only 2.8% of the volume was contributed by the pgRNA in Cp183RNA-SSS whereas 6.1% of the volume was contributed by the pgRNA in Cp183RNA-EEE. The faster disappearance of the pgRNA in Cp183RNA-SSS compared to Cp183RNA-EEE suggested that the pgRNA in Cp183RNA-SSS is more disordered though a similar result could occur if the dataset was contaminated by a large fraction of empty particles. (DOCX) [file ppat.1002919.s008.docx]

**Table S1. Relative volume calculation in Cp183_RNA_-SSS and Cp183_RNA_-EEE.**

|  | **Cp183_RNA_-SSS** | | **Cp183_RNA_-EEE** | |
| --- | --- | --- | --- | --- |
|  | **Capsid** | **pgRNA** | **Capsid** | **pgRNA** |
| **Volume rendered at 100% contour** | 84.9% | 15.1% | 84.9% | 15.1% |
| **Volume rendered at 50% contour** | 47.2% | 2.8% | 43.9% | 6.1% |

The estimated volumes for the protein shell and encapsidated RNA were calculated using the “Measure Volume and Area” function in chimera. The pgRNA volume was measured using the difference map of pgRNA calculated by subtracting empty from RNA-filled forms of SSS and EEE capsids, respectively. The contour level of the whole particle (capsid + pgRNA) and the pgRNA difference maps were first rendered at the 100% of the expected mass so that the pgRNA accounted for 15.1% of the total volume. Then the same structures were contoured at 50% of that volume. If the RNA density were as ordered as the protein density then at this contour we would expect that the RNA volume would be about 7.6% of the total volume at 100% contour. However, we observed that only 2.8% of the “100% contour volume” was attributable to pgRNA in Cp183_RNA_-SSS while 6.1% of the volume was found in Cp183_RNA_-EEE. Thus, in Cp183-EEE the RNA density is almost as ordered as the protein density. In Cp183-SSS the RNA density falls off much more steeply as a function of contour then expected. This observation suggests that the pgRNA structure in Cp183_RNA_-SSS is more disordered than in EEE. If the dataset for SSS were contaminated by a large fraction of empty particles, the volume of RNA at 100% contour would be lower than expected.
